# Interleukin-1β levels predict long-term mortality and need for heart transplantation in ambulatory patients affected by idiopathic dilated cardiomyopathy

**DOI:** 10.18632/oncotarget.15349

**Published:** 2017-02-15

**Authors:** Aneta Aleksova, Antonio Paolo Beltrami, Cosimo Carriere, Giulia Barbati, Pierluigi Lesizza, Martina Perrieri-Montanino, Miriam Isola, Piero Gentile, Elisabetta Salvioni, Tarcisio Not, Piergiuseppe Agostoni, Gianfranco Sinagra

**Affiliations:** ^1^ Cardiovascular Department, Azienda Sanitaria Universitaria Integrata di Trieste and University of Trieste, Trieste, Italy; ^2^ Department of Medical and Biological Sciences, University of Udine, Italy; ^3^ Centro Cardiologico Monzino, IRCCS, Milan, Italy, Department of Clinical Sciences and Community Health, University of Milan, Milan, Italy; ^4^ Institute for Maternal and Child Health, IRCCS “Burlo Garofolo” Trieste and University of Trieste, Trieste, Italy

**Keywords:** idiopathic dilated cardiomyopathy, long-term outcome, heart failure, interleukin 1 beta, brain natriuretic peptide

## Abstract

**Aims:**

The prognostic stratification of patients with Idiopathic Dilated Cardiomyopathy (iDCM) is a difficult task. Here, we assessed the additive value of the evaluation of biomarkers of inflammasome activation and systemic inflammation for the long-term risk stratification of iDCM patients.

**Methods and Results:**

We studied 156 ambulatory iDCM patients (mean age 58 years, 77% men, 79% in NYHA class 1-2, median Left Ventricular Ejection Fraction (LVEF) 35%, mean sodium 139 mEq/L, median BNP 189 pg/mL, median IL-1 beta (IL-1β) 1.08 pg/mL, median IL-6 1.7 pg/mL and median IL-10 2.7 pg/mL).

During the follow-up period of 89.6 months, 35 patients (22%) died/underwent heart transplantation. Patients who died/underwent heart transplantation were more likely to be male, to be in NYHA class III, to have atrial fibrillation, to have lower LVEF and higher BNP levels. IL-1β, IL-6 and IL-10 levels did not differ significantly between the groups of patients with good or bad prognosis. IL-1β levels did not vary significantly among either the different NYHA classes or the quartiles of LVEF. In a multivariable model, however, IL-1β was a strong and independent predictor of all-cause mortality (HR 1.193, 95% CI 1.056 – 1.349, p=0.005 for log squared transformed values). Other factors associated with the outcome were: male gender, presence of atrial fibrillation and sodium concentration. The estimated time-dependent ROC curve of the multivariable model showed an AUC 0.74 (95% CI 0.65-0.86).

**Conclusions:**

Serum levels of IL-1β could be useful to predict the long-term outcome of patients with iDCM.

## INTRODUCTION

Idiopathic Dilated Cardiomyopathy (iDCM) is the most frequent indication for cardiac transplantation [1]. Being more prevalent in young adults, iDCM impairs the most productive working population and, due to its morbidity and frequent and prolonged requirement for hospitalization, it is tremendously expensive for both the national health system and the society in general [2].

Physicians need to counsel patients about prognosis to enable informed decisions about medication and life expectancy. However, given the heterogeneity of the pathogenic mechanisms that lead to cardiac remodeling and heart failure, emerging mechanisms responsible for disease progression may help to identify and validate helpful biomarkers to prognostically stratify patients. In this regard, the sterile inflammatory response that occurs as a result of the activation of the inflammasome is an emerging mechanism responsible for promoting cardiac remodeling and heart failure (reviewed in [3]). Importantly, both experimental models and clinical studies have shown the involvement of the inflammasome in the evolution of both ischemic and non-ischemic heart disease. In line, a recent small clinical study suggests that the evaluation of the activation status of the NLRP3 inflammasome may be employed to evaluate the short-term outcome of patients affected by iDCM [4].

However, no literature data are available that evaluated the impact exerted by inflammasome activation on the long-term prognosis of iDCM patients. Therefore, we sought to assess the additive value of the evaluation of plasma biomarkers of inflammasome activation and systemic inflammation on top of traditional clinical variables for risk stratification of long-term mortality/heart transplantation in patients with heart failure affected by iDCM.

## RESULTS

### Study population

Patients had a median age of 58 years, 77% were male, and only 21% were in NYHA class III (being the remaining 79% in NYHA class I-II). Table 1 shows the demographic, clinical, and echocardiographic characteristics of 156 patients including the treatment, stratified by cardiac transplantation/mortality during the follow-up.

**Table 1:** Baseline characteristics of patients stratified by death/cardiac transplantation during the follow-up

	All patients(n= 156)	Alive(78%; n=121)	M/HTx(22%; n=35)	*p value*
**Age (years)**	58 (50-67)	56 (50-63)	51 (37-61)	0.34
**Male gender, n (%)**	120 (77)	88 (73)	32 (91)	0.021
**BMI (Kg/mq)**	26 (24-29)	26 (24-29)	25 (23-30)	0.56
**SBP (mmHg)**	121 ± 17	122 ± 18	120 ± 13	0.67
**DBP (mmHg)**	78 ± 14	78 ± 15	77 ± 8	0.56
**Heart Rate (bpm)**	71 ± 14	70 ± 15	71 ± 10	0.82
**Diabetic disease, n (%)**	14 (9)	9 (7.4)	5 (14)	0.21
**COPD, n (%)**	13 (8.3)	8 (6.6)	5 (14.3)	0.15
**Hb (g/dl)**	14 (13-15)	14 (13.4-15)	14.4 (12.5-15.5)	0.69
**Sodium (mEq/l)**	139 ± 3	139 ± 3	138 ± 3	0.07
**Creatinine (mg/dl)**	1.12 ± 0.34	1.09 ± 0.26	1.24 ± 0.51	0.02
**GFR, MDRD (ml/min/1.73 m^2^)**	71 (59-91)	78 (60-91)	66 (59-93)	0.85
**GFR ≤ 60 ml/min (%)**	43 (28)	28 (23)	15 (43)	0.022
**Urea (mg/dl)**	42 ± 14	42 ± 15	42 ± 9	0.97
**NYHA III (%)**	33 (21)	21 (17)	12 (34)	0.031
**Atrial fibrillation, n (%)**	19 (12)	11 (9.1)	8 (23)	0.03
**LBBB, n (%)**	62 (40)	46 (38)	16 (46)	0.41
**LA diameter index mm/ m^2^**	22 (19-25)	21 (18-24)	22 (18-26)	0.15
**LA index Area (cm^2^)**	12 (11-14)	12 (11-14)	14 (10-16)	0.32
**LV EDD index (mm/ m^2^)**	32 (28-36)	33 (29-36)	33 (30-39)	0.96
**LV ESD index (mm/ m^2^)**	25 (22-30)	25 (21-29)	28 (23-34)	0.009
**LV EDV index (ml/ m^2^)**	90 (67-106)	69 (55-98)	84 (67-104)	0.56
**LV ESV index (ml/m^2^)**	61 (39-75)	43 (30-65)	58 (36-73)	0.08
**LVEF (%)**	35 (27-42)	38 (31-46)	35 (24-46)	0.035
**MR moderate-severe n (%)**	77 (49)	57 (47)	20 (57)	0.29
**ACE inhibitors/ARBs, n (%)**	146 (94)	112 (93)	34 (97)	0.33
**Beta-blockers, n (%)**	146 (94)	112 (93)	34 (97)	0.33
**Diuretics, n (%)**	117 (75)	89 (74)	28 (80)	0.44
**Antiarrhythmics n (%)**	62 (40)	47 (39)	15 (43)	0.67
**Amiodarone n (%)**	22 (14)	15 (12)	7 (20)	0.25
**Digoxin, n (%)**	56 (36)	42 (35)	14 (40)	0.57
**Antiplatelet, n (%)**	53 (34)	41 (34)	12 (34)	0.96
**Anticoagulant, n (%)**	44 (28)	30 (25)	14 (40)	0.08
**Hypolipidemic drugs, n (%)**	51 (33)	43 (35)	8 (23)	0.16
**Nitrates, n (%)**	10 (6.4)	8 (6.6)	2 (5.7)	0.85
**Oral antidiabetics, n (%)**	7 (7.1)	5 (6.4)	2 (10)	0.58
**ICD, n (%)**	73 (47)	57 (47)	16 (46)	0.88
**CRT, n (%)**	32 (20)	23 (19)	9 (26)	0.39
**CRT-D, n (%)**	83 (53)	63 (52)	20 (57)	0.6
**BNP (pg/ml)**	189 (84-357)	181 (55-311)	258 (43-422)	0.004
**IL-1 β (pg/mL)**	1.08 (0.35-2.4)	0.77 (0.17-1.9)	0.67 (0.4-4.1)	0.38
**IL-6 (pg/mL)**	1.7 (0.6-4.4)	0.77 (0.14 −3.1)	1.5 (0.5-8.4)	0.18
**IL-10 (pg/mL)**	2.7 (0.5-11)	3 (0.8 – 9.1)	3.1 (1.4 -12)	0.34

Abbreviations: ACEI/ARBs: angiotensin converting enzyme inhibitors/angiotensin receptor blockers; BMI: body mass index; BNP: B-type natriuretic peptide; COPD: chronic obstructive pulmonary disease; CRT: cardiac resynchronization therapy; CRT-D: cardiac resynchronization therapy defibrillator; DBP: diastolic blood pressure; EDD: end-diastolic diameter; EDV: end-diastolic volume; ESD: end-systolic diameter; ESV: end-systolic volume; GFR: glomerular filtration rate; Hb: haemoglobin; ICD: implantable cardioverter defibrillator; IL: interleukin; LA: left atrium; LBBB: left bundle branch block; LV: left ventricular; LVEF: left ventricular ejection fraction; MDRD: Modification of Diet in Renal Disease; MR: mitral regurgitation; M/HTx: death/cardiac transplantation; NYHA: New York Heart Association; SBP: systolic blood pressure.

### Biomarkers correlation with clinical and laboratory variables

With regard to IL-1β, we observed significant negative correlations between this cytokine levels and both LV mass (ρ=-0.21, p=0.037) and the presence of moderate-severe mitral valve regurgitation (ρ=-0.18, p=0.023).

Additionally, BNP plasma concentration positively correlated with that of IL-6 (ρ=0.27, p=0.001).

Besides being positively correlated with BNP, IL-6 was significantly positively correlated with the NYHA III functional class (ρ=0.20, p=0.012), and both with the LVEDV index (ρ=0.19, p=0.016) and the LVESV index (ρ=0.21, p=0.007). As opposed, IL-6 levels were negatively correlated with LVEF (ρ=-0.25, p=0.002).

Finally, IL-10 concentrations did not correlate significantly with any of the variables that we evaluated.

### Long-term predictors of outcome

During the follow-up period (median 89.6 months, IQR 73.2-108.9), 35 patients (22%) died or underwent heart transplantation (31 deaths and 4 cardiac transplantations). Patients who died/underwent heart transplantation during the follow-up were more likely to be male, in NYHA class III, with atrial fibrillation and lower LV ejection fraction. The univariable analysis showed that these variables were even long-term predictors of outcome (Table 2). Among the biomarkers, BNP levels were significantly lower in survivors/non-transplanted patients versus patients who died/underwent cardiac transplantation. Importantly, we did not find a statistically significant difference in IL-1β levels between the groups that showed a different outcome during the follow-up (Table 1). However, BNP was not identified as a long-term predictor of outcome at the univariable analysis. On the contrary, IL-1β, while not having significantly different levels in the two groups of patients, was identified as a highly significant long-term predictor of outcome at univariate analysis (Table 2).

**Table 2:** Univariate and multivariate analysis of factors correlated with long-term outcome

	Univariate analysis		Multivariate analysis	
	HR (95% C.I.)	p value	HR (95% C.I.)	p value
**Age**	0.994 (0.971 – 1.018)	0.629	-	-
**Male gender**	3.571 (1.093 – 11.670)	0.035	3.351 (1.013 – 11.089)	0.048
**BMI**	0.995 (0.920 – 1-076)	0.906	-	-
**NYHA**	1.462 (0.960 – 2.227)	0.076	-	-
**NYHA III**	2.343 (1.162 – 4.724)	0.017	-	-
**SPB**	0.995 (0.975 – 1.014)	0.595	-	-
**HR**	1.002 (0.979 – 1.026)	0.842	-	-
**Atrial fibrillation**	2.634 (1.194 – 5.811)	0.016	2.564 (1.143 – 5.752)	0.022
**LBBB**	1.355 80.695 – 2.641)	0.372	-	-
**Hb**	0.962 (0.787 – 1.177)	0.709	-	-
**Anemia**	2.222 (1.083 – 4.557)	0.029	-	-
**Creatinine**	2.007 (1.082 – 3.512)	0.027	-	-
**GFR MDRD**	0.994 (0.978 – 1.010)	0.464	-	-
**GFR ≤ 60 ml/min**	2.206 (1.129 – 4.311)	0.021	-	-
**Sodium**	0.905 (0.812 – 1.009)	0.072	0.893 (0.798 – 0.999)	0.048
**LV EDD index**	1.011 (0.949 – 1.076)	0.745	-	-
**LVEF**	0.968 (0.937 – 0.999)	0.044	-	-
**MR**	1.184 (0.841 – 1.667)	0.333	-	-
**Moderate-severe MR**	1.509 (0.770 – 2.956)	0.230	-	-
**ACE inhibitors/ARB**	2.422 (0.332 – 17.699)	0.383	-	-
**Beta – blockers**	2.389 (0.327 – 17.454)	0.391	-	-
**ICD**	0.949 (0.488 – 1.846)	0.878	-	-
**CRT**	1.449 (0.679 – 3.094)	0.338	-	-
**CRT-D**	1.234 (0.631 – 2.411)	0.539	-	-
**BNP**	1.001 (1.000 – 1.001)	0.135	-	-
**IL – 1β**	1.069 (1.028 – 1.113)	0.001	1.193 (1.056 – 1.349)**	0.005
**IL – 6**	1.000 (0.998 – 1.002)	0.991	-	-
**IL – 10**	1.000 (0.998 – 1.001)	0.650	-	-

Abbreviations: ACEI/ARBs: angiotensin converting enzyme inhibitors/angiotensin receptor blockers; BMI: body mass index; BNP: B-type natriuretic peptide; CRT: cardiac resynchronization therapy; CRT-D: cardiac resynchronization therapy defibrillator; EDD: end-diastolic diameter; GFR: glomerular filtration rate; Hb: haemoglobin; ICD: implantable cardioverter defibrillator; IL: interleukin; LBBB: left bundle branch block; LV: left ventricular; LVEF: left ventricular ejection fraction; MDRD: Modification of Diet in Renal Disease; MR: mitral regurgitation;; NYHA: New York Heart Association; SBP: systolic blood pressure.

**log squared transformed values.

**Figure 1:**
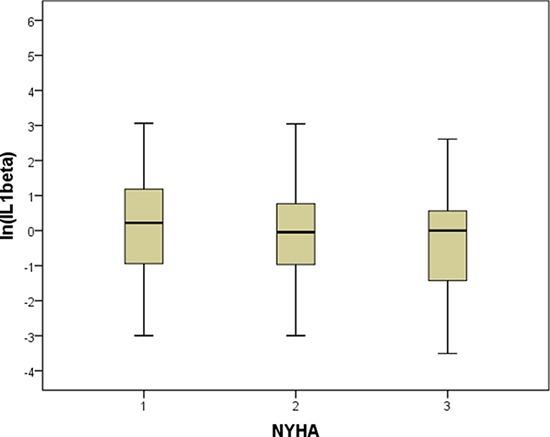
Levels of IL-1β in patients with NYHA functional class I to III The bottom and top of the box represent the first and third quartiles, and the band inside the box is the median. Lines extending vertically from the boxes (whiskers) indicate variability outside the upper and lower quartiles, i.e. respectively the lowest value still within 1.5 IQR (interquartile range) of the lower quartile, and the highest value still within 1.5 IQR of the upper quartile.

**Figure 2:**
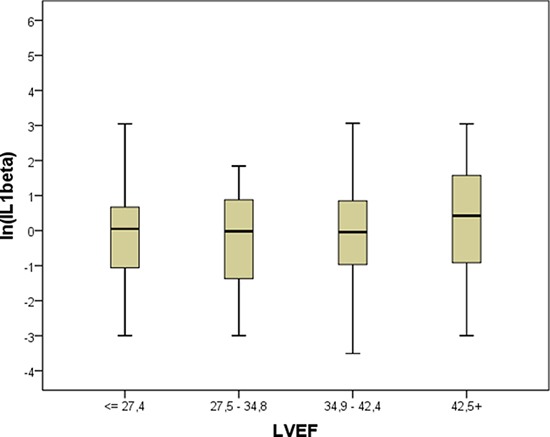
Levels of IL-1β in patients as function of quartiles of LV ejection fraction As in Figure 1, box plots of IL-1β values are depicted according to quartiles of LV ejection fraction. See Figure 1 legend for box plot explanation.

**Figure 3:**
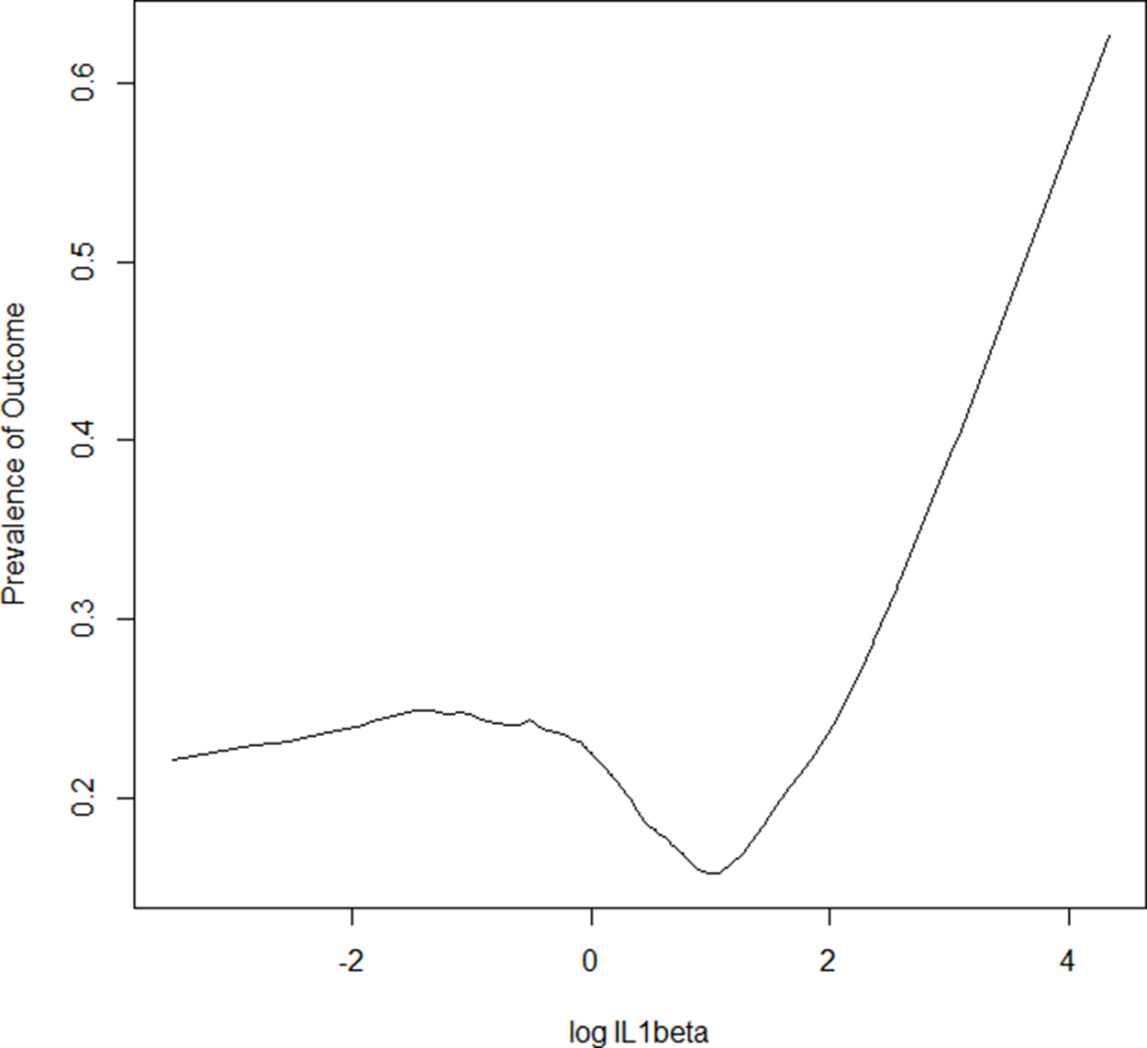
Relationship between IL-1β concentration and outcome Plot of smoothed estimates of (natural) log values of IL-1β concentration on the x-axis vs. rates of outcome on the y-axis. The “plsmo” function of the R library “Hmisc” has been used to generate the plot; locally-weighted polynomial regression is used to generate the depicted curve.

We did not observe a significant correlation between BNP and IL-1β, suggesting that these biomarkers assess different aspects of the heart failure syndrome. IL-1β levels did not vary significantly among either the different NYHA classes or the quartiles of LVEF (Figures 1 and 2). In order to visualize the relationship between IL-1β concentration and the prevalence of the composite outcome, we fitted a smoothed curve between logarithmic values of IL-1β and the frequency of the outcome, reported in Figure 3. We observed a low prevalence of outcome at low concentrations of ln (IL-1β) and then a steep increase in prevalence starting from 1 for ln (IL-1β). For this reason, we inserted in the final multivariable model a transformed version of ln (IL-1β), set at 0 for ln (IL-1β) < 1, i.e. for original values of IL-1β lower than 2.7, and set at ln (IL-1β)**2 (square) for values greater or equal than 2.7.

**Figure 4:**
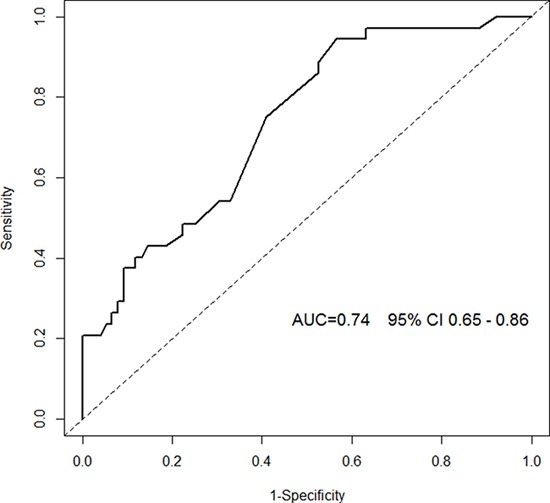
Time-dependent ROC curve of the multivariable model Area under the Curve (AUC) with corresponding 95% CI (confidence interval) is depicted: to calculate AUC we used as a marker the risk of event estimated by the multivariable model and as time-horizon events within 96 months of follow up. Inverse Probability of Censoring Weighting (IPCW) is used to account for the censoring mechanism. Technical details about time-dependent ROC computation can be found in [20].

In the final multivariable model (that includes clinically relevant variables as well as those variables with p < 0.1 at univariate analysis) were independently associated with long term mortality/cardiac transplantation the following variables: male gender, presence of atrial fibrillation, plasma sodium concentration and the transformed logarithmic squared version of IL-1β values (Table 2). The estimated time-dependent ROC curve of the multivariable model showed an AUC 0.74 (95% CI 0.65-0.86) with an horizon of the events fixed at 96 months (Figure 4).

## DISCUSSION

This report represents the multicenter observational study with the largest cohort of real-life outpatients affected by heart failure secondary to iDCM in whom the long-term impact of inflammasome activation on mortality/need for cardiac transplantation has been described. The main finding of this study is that the prognostic information provided by the measurement of IL-1β plasma levels is independent of and beyond that provided by other relevant clinical factors, including the left ventricular ejection fraction as well as the degree of neurohumoral activation (sodium levels and plasma BNP).

The progression of heart failure is complex and is driven by multiple biologic processes, such as neurohormonal activation and inflammation.

As introduced, an accumulating body of literature demonstrates the primary role played by inflammasome activation in the evolution of heart disease towards cardiac remodeling and heart failure. A recent work by Luo and collaborators has recently measured inflammasome components in peripheral blood mononuclear cells and IL-1β in the plasma of 54 iDCM patients and 20 healthy controls. The study showed that NLRP3 and IL-1β mRNA levels are independent predictors of the necessity for rehospitalization in a six month follow-up [4]. The current study significantly expands the findings of the previous cited study in ambulatory setting of larger cohort of patients with iDCM and with long-term follow-up. Our findings, that associate IL-1β levels with patient mortality/necessity for transplantation, may have a therapeutic importance too, since the inhibition of the inflammasome/IL-1β axis is being investigated as a promising target [5]. In line, a single intraperitoneal injection of IL-1β is able to repress the systolic function of healthy mice in a dose-dependent fashion. Furthermore, the addition of Anakinra (the soluble IL-1 receptor antagonist) to IL-1β fully prevented systolic dysfunction [6].

With regard to possible comorbidities that may influence IL-1β levels diabetes [7], myocarditis [8] and other autoimmune diseases [9] are among the most investigated ones. In our case study, the frequency of diabetic patients did not differ among the groups with different outcome, nor it was significantly associated with outcome at univariate analysis. Furthermore, patients suffering from acute myocarditis or known autoimmune diseases were excluded from our analysis.

Along with IL-1β, we investigated also whether IL-6 plasma levels are able to predict the prognosis of patients affected by iDCM. IL-6 is a pleiotropic cytokine that has been shown to exert both protective actions (e.g. favoring the resolution of the neutrophilic infiltrate) and to participate to disease pathogenesis (e.g. promoting the monocyte infiltration). Studies conducted on iDCM patients have shown increased production of IL-6 [10]. However, in the iDCM setting, IL-6 levels are not clearly associated with left ventricular reverse remodeling or patients’ prognosis [11]. Our study is in line with these observations and shows that, although IL-6 levels are significantly correlated with important clinical parameters, it is not able to predict long term prognosis of patients.

In addition to inflammatory processes, the complex pathways of neurohormonal activation are observed and their quantification through biomarkers might relay important information about heart failure. In our study, serum sodium levels were independently associated with the long-term outcome. In the setting of heart failure, hyponatremia reflects more severe activation of the renin–angiotensin–aldosterone and of the sympathetic nervous system as well as increased vasopressin release [12]. Also, hyponatriemia could be associated with the diuretic therapy, which enhances sodium excretion [12]. A recent meta-analysis [13], that included 14,766 patients from 22 studies and used as endpoint the death from any cause at three years, showed that the risk of death is linearly increasing with serum sodium levels < 140 mmol/L. Natriuretic peptides, indicative of myocardial stretch, are well-recognized prognostic biomarkers in the setting of heart failure [14]. In our study, despite we observed significantly higher BNP levels at baseline in patients who died/underwent heart transplantation, BNP was not an independent predictor of long-term outcome at multivariate analysis; however the median levels of BNP in both groups of our population were under the diagnostic threshold for heart failure. Several studies found that BNP levels are not always elevated, and the paradoxically low BNP level is an adverse prognostic marker in patients with advanced heart failure [15–17]. In the setting of patients with chronic stable heart failure, one recent systematic review [18] does not recommend to clinicians BNP or NT-proBNP use for risk stratification of patients.

### Study limitation

The present study focused primarily on the long-term mortality/need for cardiac transplantation of patients with iDCM. We accept as a limitation that we could not provide information on the time-course of plasma biomarker levels since the blood sample for biomarker analysis was drawn only at baseline.

In conclusion, serum levels of IL-1β were able to prognostically stratify our cohort of iDCM patients. The precise cut-off of IL-1β associated with bad outcome in iDCM remains to be determined in larger, independent cohorts of patients.

## MATERIALS AND METHODS

A cohort of 156 patients with iDCM referred to Cardiology of Trieste and Monzino was prospectively enrolled between March 30^th^, 2006 and January 31^st^, 2010 (iDCM is a rare heart muscle disease with an incidence of 5 to 8 cases per 100,000 persons-year). The diagnosis of iDCM was made according to the World Health Organization criteria [19].

Clinical and instrumental data were collected at the moment of enrolment and during each follow-up visit. Patients had schedule of visits at six, 12, 24 months and, subsequently, every two years or more frequently on the base of specific clinical needs.

Blood samples were collected in ethylenediaminetetraacetic acid, immediately placed on ice, processed, and stored at -80°C until analysis was performed. The same blood sample was employed to test the concentrations of B-type natriuretic peptide (BNP), IL-1β, Interleukin 6 (IL-6 ) and Interleukin 10 (IL-10). We tested BNP also, since natriuretic peptides are currently the benchmark against which all biomarkers for prognosis of patients with heart failure must be measured.

### Determination of biomarkers

The quantitative determination of the BNP was measured from plasma samples obtained by centrifuging whole blood samples at 2,500g for 10′ at 4°C. Plasma levels of BNP were assayed using the competitive ELISA immunoassay RayBio (r) BNP Enzyme Immunoassay (RayBiotech, Inc., Norcross, GA), according to the manufacturer's protocol. The minimum detectable concentration of BNP was: 1.66 pg/ml with an intra-assay coefficient of variation (CV) of < 10 % and an inter-assay CV of < 15 %.

For the determination of plasma levels of IL-1 β, a high-sensitivity ELISA kit (HS Quantikine Human IL-1 β Immunoassay, R&D Systems, Inc, MN, USA) has been used. The minimum detectable dose of Il-1β ranged from 0.023 to 0.140 pg/mL. The mean minimum detectable dose was 0,057 pg/mL. The concentration of IL-1β has been measured by subtracting the readings at 650nm from the readings at 450nm at the spectrophotometer.

IL-6 High sensitivity sandwich ELISA (Quantikine HS, R&D Systems, Minneapolis, MN) was used to measure IL-6 plasma levels. The minimum detectable dose was of 0.11 pg/mL while the kit's range was 0.2 - 10 pg/mL.

Plasma levels of IL-10 were measured with The Quantikine Human IL-10 Immunoassay solid phase ELISA (R&D Systems, Inc, MN, USA). This immunoassay has been shown to quantify recombinant human IL-10 and does not cross-react with viral IL-10. The optical density was determined at 450nm with wavelength correction at 570 nm by using a microplate reader. The sensitivity of the test of human IL-10 was less than 3.9 pg/mL.

### Follow-up and outcomes

The main endpoint was all-cause mortality/urgent heart transplantation. For the objective of our study urgent heart transplantation was considered in status I, indicated in patients with refractory heart failure with necessity of inotropic treatment and/or mechanical support of the circulation.

Information regarding the end-points were obtained directly from the patient, from their family doctor or from the registers of death of the communes of residence. The end of the follow-up was set to December 31^st^, 2015 or the date of death or heart transplantation of the patient. This study conforms to the principles outlined in the Declaration of Helsinki and was approved by the institutional ethics committee. All patients provided written informed consent prior to enlistment in the Registry.

### Statistical analysis

Characteristics of the study population are described using means ± s.d. or median and interquartile range for continuous variables, depending on the distribution's shape, and percentages for categorical variables. Data were tested for normal distribution using the Kolmogorov-Smirnov test. T-test or Mann-Whitney test, as appropriate, were used to compare continuous variables between two groups. For categorical variables, cross-tabulations were generated, and chi-square or Fisher exact test was used to compare distributions.

To test the capacity of the biohumoral parameters to prognostically stratify iDCM patients, we analyzed death or necessity of cardiac transplantation as endpoint. Cox univariable analysis was performed and clinically relevant covariates as well as those with p < 0.1 were selected for multivariable analysis. Retention in the final stepwise model required the variable to be significant at p < 0.05 at multivariate analysis. Results are presented as hazard ratios (HR) and 95% confidence intervals (95% CI). The proportional hazards assumption of the Cox model was checked using the Therneau and Grambsch test.

Since distributions of the biohumoral parameters showed a marked asymmetry, we performed a logarithmic transformation of their values and repeated the stepwise procedure using the log-transformed values. Log transformed data confirmed our findings on mortality/cardiac transplantation.

Finally, the obtained score from the multivariable model was evaluated in terms of predictive accuracy by means of time-dependent ROC curve suitable for censored data [20]. Analyses were conducted with IBM-SPSS 21 statistical software and the R package version 3.2.2, using libraries “survival”, “rms”, “Hmisc” and “timeROC”.

## Abbreviations

AUC: Area Under Curve
BNP: Brain Natriuretic Peptide
CI: Confidence Interval
HR: Hazard Ratio
iDCM: Idiopathic Dilated Cardiomyopathy
IL: Interleukin
IQR: Interquartile Range
LV: Left Ventricle
LVEDV: Left Ventricular End-Diastolic Volume
LVEF: Left Ventricular Ejection Fraction
LVESV: Left Ventricular End-Systolic Volume
NLRP3: Nucleotide-Binding Oligomerization Domain, Leucine Rich Repeat And Pyrin Domain Containing 3
NYHA: New York Heart Association
ROC: Receiver Operating Characteristics.
